# Long-term outcomes following lower extremity press-fit bone-anchored prosthesis surgery: a 5-year longitudinal study protocol

**DOI:** 10.1186/s12891-016-1341-z

**Published:** 2016-11-22

**Authors:** Ruud A. Leijendekkers, J. Bart Staal, Gerben van Hinte, Jan Paul Frölke, Hendrik van de Meent, Femke Atsma, Maria W. G. Nijhuis-van der Sanden, Thomas J. Hoogeboom

**Affiliations:** 1 Department of Orthopaedics, Physical Therapy, Radboud university medical centre, Geert Grooteplein-Zuid 10, 6525GA Nijmegen, The Netherlands; 2 Master Clinical Health Sciences, Program in Physical Therapy Science, University Utrecht and University Medical Centre Utrecht, Utrecht, The Netherlands; 3 Radboud Institute for Health Sciences, IQ healthcare, Radboud university medical centre, Nijmegen, The Netherlands; 4 Research group Musculoskeletal Rehabilitation, HAN University of Applied Sciences, Nijmegen, The Netherlands; 5 Department of Surgery, Radboud university medical centre, Nijmegen, The Netherlands; 6 Department of Rehabilitation, Radboud university medical centre, Nijmegen, The Netherlands

**Keywords:** Amputees, Artificial limbs, Osseointegration, Functional outcomes

## Abstract

**Background:**

Patients with lower extremity amputation frequently suffer from socket-related problems. This seriously limits prosthesis use, level of activity and health-related quality of life (HRQoL). An additional problem in patients with lower extremity amputation are asymmetries in gait kinematics possibly accounting for back pain. Bone-anchored prostheses (BAPs) are a possible solution for socket-related problems. Knowledge concerning the level of function, activity and HRQoL after surgery is limited.

The aims of this ongoing study are to: a) describe changes in the level of function, activity, HRQoL and satisfaction over time compared to baseline before surgery; b) examine potential predictors for changes in kinematics, prosthetic use, walking ability, HRQoL, prosthesis comfort over time and level of stump pain at follow-up; c) examine potential mechanisms for change of back pain over time by identifying determinants, moderators and mediators.

**Methods/design:**

A prospective 5-year longitudinal study with multiple follow-ups. All adults, between May 2014 and May 2018, with lower extremity amputation receiving a press-fit BAP are enrolled consecutively. Patients with socket-related problems and trauma, tumour resection or stable vascular disease as cause of primary amputation will be included. Exclusion criteria are severe cognitive or psychiatric disorders. Follow-ups are planned at six-months, one-, two- and five-years after BAP surgery. The main study outcomes follow, in part, the ICF classification: a) level of function defined as kinematics in coronal plane, hip abductor strength, prosthetic use, back pain and stump pain; b) level of activity defined as mobility level and walking ability; c) HRQoL; d) satisfaction defined as prosthesis comfort and global perceived effect. Changes over time for the continuous outcomes and the dichotomized outcome (back pain) will be analysed using generalised estimating equations (GEE). Multivariate GEE will be used to identify potential predictors for change of coronal plane kinematics, prosthetic use, walking ability, HRQoL, prosthesis comfort and for the level of post-operative stump pain. Finally, potential mechanisms for change in back pain frequency will be explored using coronal plane kinematics as a potential determinant, stump pain as moderator and hip abductor strength as mediator.

**Discussion:**

This study may identify predictors for clinically relevant outcome measures.

**Trial registration:**

NTR5776. Registered 11 March 2016, retrospectively registered.

**Electronic supplementary material:**

The online version of this article (doi:10.1186/s12891-016-1341-z) contains supplementary material, which is available to authorized users.

## Background

The population living with a lower extremity amputation is estimated to grow significantly, in part, due to the aging population and high rates of vascular disease [1]. In the Netherlands, 90–94% of the lower extremity amputations are due to vascular disease, 3% to trauma and 3% to tumour resection [2]. The primary amputation level is transtibial in 49% of the patients, knee disarticulation in 9%, transfemoral in 34% and bilateral in 9% [3]. Approximately 86% of the patients with a lower extremity amputation are fitted with a socket prosthesis (SP) [4]. Within socket prosthesis users, 34–63% have reported suffering from socket-related problems including chronic skin problems and residual limb pain associated with the socket [5–9]. It is well known that the symmetry in spatio-temporal, kinematic and kinetic parameters during gait is reduced in SP users compared to able-bodied persons [10–12]. In addition socket fitting problems [13], decreased hip abductor strength [13–15] and changed muscle activity patterns [16] may be a possible cause for this asymmetry. Gait asymmetry, specifically in the coronal [17–19] and sagittal plane [18], are considered to be associated with secondary complaints such as back pain [20]. Both, socket-related problems and back pain can lead to limited prosthetic use [5, 21] and reduced health-related quality of life (HRQoL) [5, 7].

For patients with a lower extremity amputation who suffer from socket-related problems the prosthesis can be transcutaneously attached to the bone by osseointegration utilizing intramedullary implants, known as bone-anchored prostheses (BAPs) [22]. BAPs are used in patients with a transfemoral [23–30] or transtibial amputation [31–33]. To date, there are two types of implants available: a screw BAP [34, 35] and a press-fit BAP [29, 36]. Both are implanted using two-step surgery techniques, however, for the press-fit BAP the time between surgeries is 4.5 months shorter than for a screw BAP [34, 36]. Additionally, the rehabilitation period is shorter for press-fit BAP because the implant allows more weight bearing in the early post-operative phase [29, 34–36].

An advantage of direct attachment to the bone, regardless of the used BAP method, is that the patient has a better ability to detect vibrotactile and pressure stimuli of the prosthetic limb compared to socket prosthesis users [22, 37, 38]. These stimuli are also known as osseoperception. Although BAP surgery has been performed for over 25 years only eight and generally small longitudinal studies [23, 25, 27–31, 39] assessed the level of function, activity and QoL outcomes of BAP compared to SP use. Various benefits have been found on body functions or structures (hereafter referred to as level of function), level of activity and HRQoL for BAP use compared to SP use [40, 41]. However, functional outcomes seem to be an underexposed part of BAP research to date. This is remarkable, because BAP surgery is an invasive intervention aimed to overcome problems in physical functioning in SP users with socket-related problems. In the SP population, complaints such as back pain may be the result of asymmetries in coronal plane gait kinematics and are possibly related to hip abductor strength deficiencies [20, 42], but have not been researched in the BAP population. Research on the level of satisfaction experienced by the patient with respect to their prosthesis is absent. Similarly, factors associated with outcomes regarding the levels of function, activity, HRQoL and satisfaction of the prosthesis after BAP surgery has not been researched. This study will address the above mentioned outcomes and follow, in part, the ICF classification [41]. The purpose of this manuscript is to detail the study protocol of an ongoing prospective 5-year longitudinal study with multiple follow-ups. The study focuses on patients with a lower extremity amputation who are fitted with a press-fit BAP and complete a standard rehabilitation programme. Through publishing this study protocol we aim to: a) increase the transparency of our data collection; b) prevent publication bias and selective reporting; c) prevent data dredging [43]. The study has four aims:

Aim 1: to describe the change in the level of function, activity, HRQoL and satisfaction in patients with a lower extremity amputation after receiving a press-fit BAP at short-term (six-months and one-year), mid-term (two-years) and long-term (five-years) follow-up in comparison to baseline. We hypothesise that coronal plane kinematics symmetry, hip abductor strength, prosthetic use, mobility level, walking ability, HRQoL and prosthesis comfort will improve over time and that the frequency of back pain will decrease over time.

Aim 2: to examine potential predictors for the change of coronal plane kinematics, prosthetic use, walking ability, HRQoL and prosthesis comfort over time: outcomes that are the main reason why patients choose a BAP The potential predictors which will be included in the analysis are: demographic data, patient characteristics, baseline level of function, baseline level of activity and baseline level of satisfaction (Additional file 1).

Aim 3: to examine predictors for the level of stump pain at short-term, mid-term and long-term follow-up (Additional file 1). In our clinic we see that stump pain after BAP surgery negatively influences outcomes on the level of function, activity, HRQoL and satisfaction. However, we do not have insight in the level of stump pain at follow-up and knowledge concerning predictors for stump pain is absent.

**Fig. 1:**
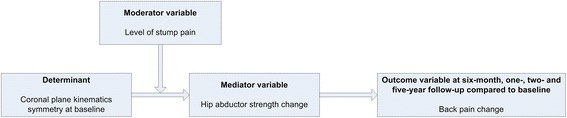
Causal model for change of back pain

Aim 4: to examine potential mechanisms for changes in back pain at short-term, mid-term and long-term follow-up. We hypothesise that an increase of coronal plane kinematics symmetry will be a possible determinant for a decrease in back pain (Fig. 1). We also hypothesise that stump pain will act as moderator and hip abductor strength will act as mediator.

The short-, mid- and long-term outcomes and the results of the various aims of this study will be presented in separate articles.

## Methods and design

This is an ongoing prospective 5-year longitudinal study with multiple follow-ups. All assessments are part of usual care for patients with a lower extremity amputation following BAP surgery.

### Study population

All patients who are eligible for a press-fit BAP in the Netherlands undergo surgery in one hospital (Radboud university medical centre). All consecutive patients in our centre, between May 2014 and May 2018, undergoing BAP surgery are eligible for this study. Patients are eligible for press-fit BAP surgery if: a) they are adults with a lower extremity amputation suffering from socket-related problems contributing to limited prosthetic use; b) the cause of primary amputation is congenital or due to a trauma, tumour resection or stable vascular disease. Exclusion criteria for surgery are the presence of severe cognitive or psychiatric disorders. A multidisciplinary team including a surgeon, rehabilitation physician, physiotherapist and prosthesist assess patients for inclusion for BAP using a standard procedure as described by Van de Meent et al. [29]. When patients’ medical history reveals a psychiatric history, a psychologist is consulted to assess the patient prior to inclusion.

### Sample size

We will not draw a sample, but aim to include the entire population, which started in May 2014. We expect to have little non-responders as all assessments are part of usual care. This expectation is supported by the fact that we had no non-responders since the start of this study. Based on the average number of surgeries in the Netherlands during the year 2014 and 2015 we expect that 18 patients will be included each year [44]. For our first aim, investigating change over time, we will present the first interim analyses of this growing cohort when we have a minimum of 40 patients at the following time points: one-, two- and five-year follow-up. Press-fit BAP surgery started in the Netherlands in 2009. Between 2009 and 2014, 42 patients received a BAP. Thus at the time of our first interim analyses (mid of 2017) the total Dutch population with a minimum follow-up of one-year will include 82 patients [44]. Although, the sample size of 40 patients appears to be small, the study will have gathered longitudinal data (with a minimum follow-up of one-year) in 49% of the total Dutch population of patients with a press-fit BAP. We expect that this is sufficient to be able to generalise the results to the Dutch population eligible for a press-fit BAP. Of the patients included in this study to date, none have dropped out. We also do not anticipate any high drop-out rates for the remainder of the study as the assessments are part of usual care.

We will use the rule-of-thumb for prediction modelling with multiple factors and assume that we need ten cases per predictor [45]. The various continuous outcomes of interest have a different number of potential predictors (Additional file 1) ranging from three to seven variables. A minimum of 30 subjects are needed for prosthetic use, 40 subjects for HRQoL, 50 subjects for walking ability and prosthesis comfort, 60 subjects for coronal plane kinematics, and 70 subjects for stump pain. Based on these numbers we expect to complete the inclusion of patients for this study around May 2018.

### Intervention

Press-fit BAP surgery involves two surgeries six to eight weeks apart [29, 36]. First, a cementless intramedullary stem is inserted in the femur or tibia and the wound is closed. After osseointegration has been initiated, a second procedure creates a soft tissue stoma with a transcutaneous connector which is bolted into the intramedullary stem. Between the two surgeries the patient is not allowed to use a socket prosthesis but is permitted to ambulate on the sound limb using a walking aid, such as crutches.

**Fig. 2:**
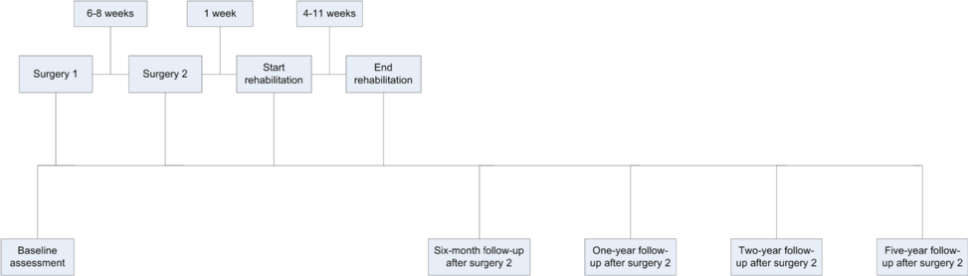
Flow chart of assessments and interventions

All patients start rehabilitation one week after the second surgery. Rehabilitation aims to reach predetermined functional goals. These goals include increasing the level of activity and minimising gait compensation strategies, such as, an unstable pelvis and ipsilateral lateral flexion of the trunk during stance phase. Rehabilitation focuses on improving hip abductor strength, core stability, symmetry in spatio-temporal parameters and symmetry in kinematic parameters. The detailed rehabilitation programme is described elsewhere [46]. The duration of the twice weekly rehabilitation programme (Fig. 2) depends on the level of amputation level and ranges from 4 weeks (transtibial amputation) to 11 weeks (transfemoral amputation). Rehabilitation is prolonged if the patient is improving but has not met the previously determined goals.

### Ethics

Patients included in this study are informed about the baseline and follow-up assessments by the treating physician during the multidisciplinary out-patient clinic for BAP. A separated document, including a patient information letter and informed consent form is attached to the baseline assessment appointment letter. Informed consent with permission to use the usual care data for research purposes is obtained prior to the baseline assessment. The study is conducted according to the principles of the Declaration of Helsinki (64th version, 19-10-2013). The protocol of this study (registration number 2014/196) was approved by the Ethics Committees of Radboud university medical centre.

### Study procedures and parameters

Patients are assessed by the treating physiotherapist at baseline (preoperatively) and at six-month, one-, two- and five-year follow-up (Fig. 2). The primary outcomes of this study are level of function, activity, HRQoL and satisfaction. Demographics and patient characteristics are obtained from the patients. These variables will be used for descriptive statistics, and some will be used as potential predictors for change of coronal plane kinematics, prosthetic use, walking ability, HRQoL, prosthesis comfort and the level of stump pain at follow-up (Additional file 1).

#### Demographics and patient characteristics

Sex, age, cause of amputation and the time from primary amputation to inclusion are collected at baseline from the patients’ medical file. Level of amputation is obtained at baseline and at six-month follow-up. Body mass index (BMI) accounting for the limb loss using the adjusted body weight [47], residual limb and sound limb characteristics [46, 48] and used prosthesis parts are obtained at baseline and all follow-up time points. Rehabilitation characteristics (the duration in weeks and the number of rehabilitation sessions) are also obtained. Adjusted body weight is calculated with the following formula: actual body weight/(1 minus percentage of the amputated part of the limb). The amputation percentages are: Transfemoral Amputation (TF): 10.1%, Transtibial Amputation (TT)/knee disarticulation (KD): 5.9%, foot amputation: 1.5%, bilateral TF: 20.2%, bilateral TT/KD: 11.8%, bilateral foot amputation: 3.0% and TF combined with TT/KD: 16.0% [47].

#### Level of function

##### Coronal plane kinematics

**Fig. 3:**
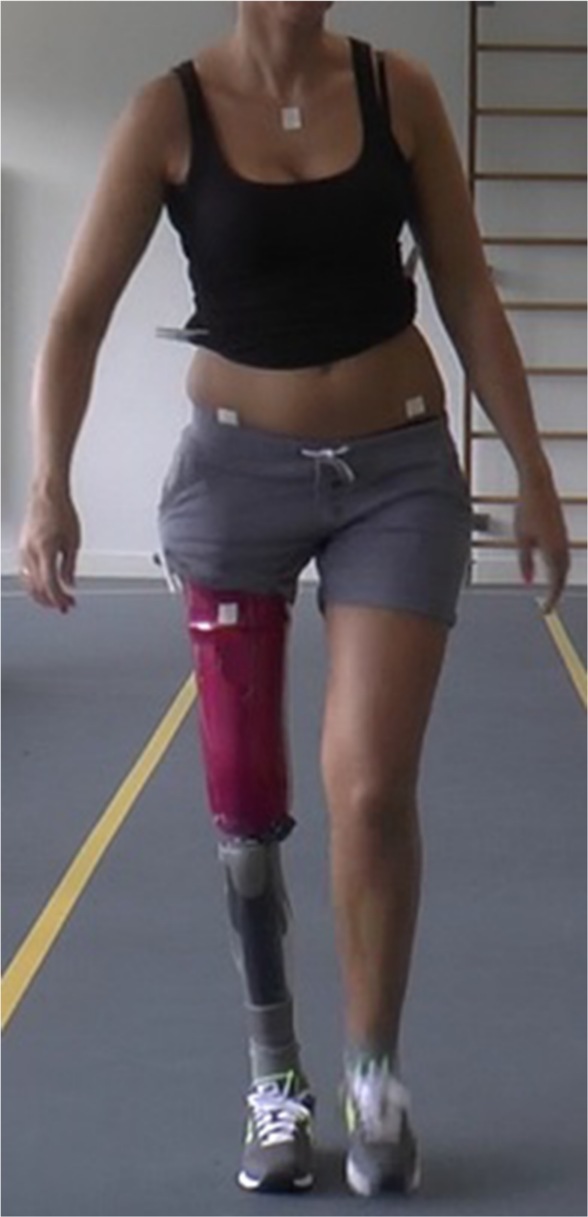
Position of the tapes

**Fig. 4:**
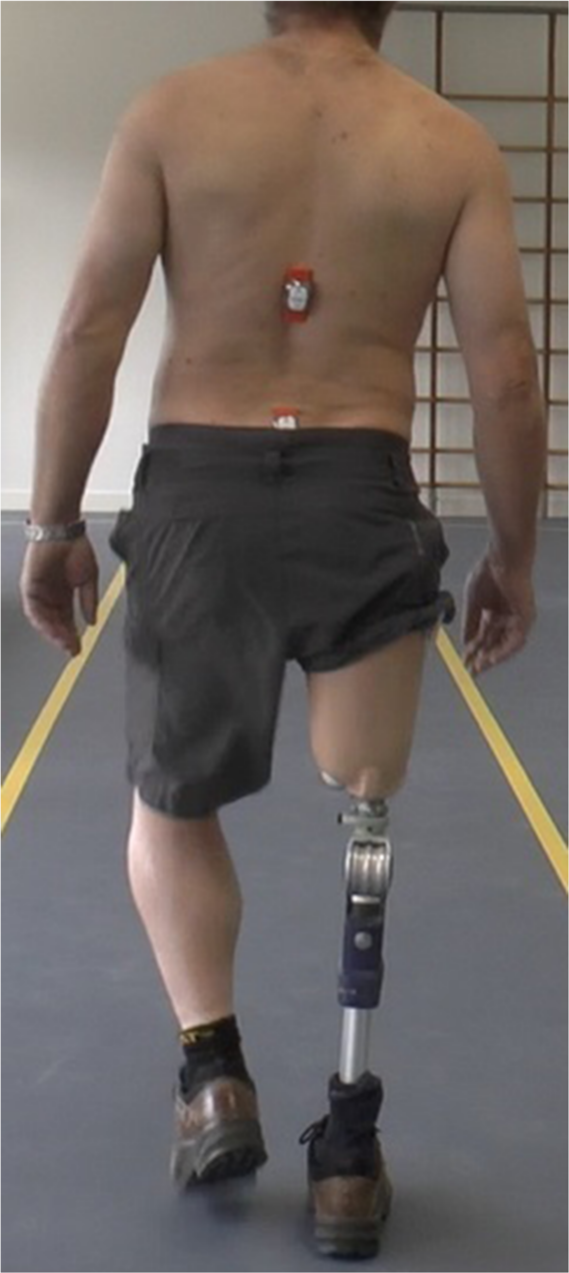
Position of the gyroscopes

In unaided walkers, kinematics in the coronal plane are recorded using a video camera (Panasonic HC-X920) during two activities. First, while a patient walks three times up and down a path of 15 m. Secondly, while a patient performs a step exercise with the sound side. The step exercise is performed, two times consecutively using a 11 cm high aerobic power step (Tunturi® New Fitness, Almere, The Netherlands). The kinematics (continuous scale) in coronal plane (in degrees) are assessed using two methods: a) an overall angle between trunk and residual limb during the mid-stance is calculated out of two angle measurements, namely the angle between pelvis and residual limb and the angle between pelvis and trunk. To be able to assess these angles using Dartfish® software (Dartfish, Fribourg, Switzerland), a piece of tape (approximately 1.0 by 1.0 cm) is placed on 1) the anterior superior iliac spine (ASIS) on both sides, 2) the proximal part of the manubrium and 3) 30 cm distal of the ASIS on the ventral side of the residual limb (Fig. 3). The reference points for the position of the tape on the residual limb varies. In patients with a transtibial amputation the middle of the patella is used as a reference for all assessments. At baseline, the middle of the socket is used in patients with transfemoral amputation or knee disarticulation, because the position of the bone in soft tissue is not visible. At follow-up the transcutaneous connector is used in patients with a transfemoral amputation; b) peak pelvis and trunk segment angles during stance phase are measured relative to the laboratory axis using two wireless gyroscopes (Valedo®Motion, Hocoma, Volketswil, Switzerland) on the first vertebrae of the sacrum and 17.5 cm cranial of the distal gyroscope respectively, (Fig. 4) using an applicator. A reproducibility study is now ongoing to assess both angle measurements and will determine which instrument will be used to evaluate the kinematics.

##### Hip abductor strength

Hip abductor strength (continuous scale) is measured using the ‘make-technique’ with a microfet2™ handheld dynamometer (HHD) (Hoggan Scientific LLC., Salt Lake City, Utah, United States) with the patient lying in supine position. A fixation-belt is used to stabilise the pelvis and prevent sliding. The HHD is applied 22 cm distal of the most prominent aspect of the greater trochanter. For each strength test, following a warm-up of one submaximal contraction, all patients perform three maximal trials for 3 to 5 s with a 1-min rest interval. The maximum of three valid trials is used. The torque value (Nm) of the hip musculature is normalised by body weight in kilograms (kg), resulting in a torque value in Nm/kg. This hip abductor strength test is a modification from the test described by Pua et al. [49]. A reproducibility study is now ongoing.

##### Prosthetic use

The prosthetic use score (continuous scale: 0–100 points) of the Dutch version of the questionnaire for persons with a transfemoral amputation (Q-TFA) is used to assess the patient-reported prosthesis wearing time [50]. A higher score represents a longer wearing time.

##### Back pain

A single question (ordinal scale) ‘*Did you experience back pain within the previous month?*’ with three response alternatives; ‘no’, ‘yes, with episodes’ and ‘yes, chronic (daily)’ is used to assess back pain frequency [46].

##### Stump pain

Post-operative stump pain (continuous scale) is assessed during follow-up using the Numeric Rating Scale (NRS: 0–10) [51]. Pain location (nominal scale) is assessed for descriptive purposes with seven response alternatives: ‘no location’, ‘soft tissue stoma’, ‘distal side stump’, ‘ventral side stump’, ‘inguinal area’, ‘greater trochanter area’ or ‘other’.

#### Level of activity

##### Mobility level

The mobility level is assessed using three patient-reported outcome measures (ordinal scales: a-c) and one physical performance measurement (continuous scale: d): a) the Medicare Functional Classification Level (MFC-level) [52], also known as ‘k-levels’ (0–4) in which ‘k0’ represents a non-ambulatory person and ‘k4’ a high-level prosthesis user; b) the Special Interest Group in Amputee Medicine Workgroup Amputation and Prosthetics (SIGAM WAP) mobility score (class A-F) [53] where ‘class A’ represents an abandoned prosthesis user and ‘class F’ a prosthesis user with a normal gait without aids; c) a question concerning the use of aids in daily life, both for indoor use and outdoor use, with a 5-point likert scale answer; ‘wheelchair-bound’, ‘walking frame/rollator’, ‘two crutches/canes’, ‘one crutch/cane’, ‘none’; d) the timed up and go (TUG) using a standard arm chair (seat height 46 cm, arm height 67 cm) and a 3 m walking course marked by a pylon, representing the level of physical mobility [54]. The fastest attempt (in seconds) of three TUGs is noted as final time score.

##### Walking ability

Walking ability is evaluated using two measurements (continuous scales): a) a self-paced 6-minute walking test (6MWT). Patients walk six minutes as fast as they can without encouragement, on a 10 m course marked by two pylons representing the submaximal level of functional capacity [55, 56]. Total walking distance will be recorded in metres and walking speed will be calculated in metres per second as an indicator of gait performance [57, 58]; b) a single question: ‘*How far can you walk in one go in everyday life?*’ representing a patient-reported estimation of the walking distance in daily life in metres [46].

#### Level of health-related quality of life

The Q-TFA global score (continuous scales: 0–100 points) is used to assess HRQoL and is a summary of three items: perception of function, problems with the current prosthesis and the perception of the current overall amputation situation [29, 50]. A higher score reflects a better HRQoL. All three items are scored on a 5-point likert scale. For patients not using a prosthesis only the single overall question (ordinal scale) is used; ‘*How would you summarize your overall situation as an amputee?*’, with five response alternatives; ‘extremely poor’, ‘poor’, ‘average’, ‘good’ or ‘extremely good’.

#### Level of satisfaction

##### Prosthesis comfort

To assess the satisfaction of the patient in regards to their prosthesis, including the socket or bone-anchored prosthesis aspect, the Prosthesis Comfort Score (PCS) [46] is used. The PCS (continuous scales) is a single question ‘*How satisfied are you with your current prosthesis on a scale of 0 to 10, where 0 is not satisfied and 10 extremely satisfied?*’.

##### BAP satisfaction

Global perceived effect of BAP (ordinal scale) is assessed within the post-operative follow-up using a single question ‘*Would you, with your current knowledge, choose for a BAP again?*’ with five response alternatives; ‘strongly disagree’, ‘disagree’, ‘neutral’, ‘agree’ or ‘strongly agree’.

### Statistical analysis

All outcomes will be analysed non-stratified. Additionally, we will stratify for level of amputation and for baseline wheelchair-boundedness when applicable. The size of the subgroups will determine whether statistical tests will be used to analyse the outcomes of the subgroups, or if only descriptives will be presented. All analyses will be performed using SPSS v23 (SPSS Inc., Chicago, Illinois, United States). In all cases, two sided *p*-values <0.05 will be considered to be statistically significant.

#### Descriptive statistics

Categorical data will be presented as exact numbers. Percentages will be calculated for the various levels. For the continuous data, means and standard deviations will be calculated for normally distributed variables. For data not-normally distributed median and inter-quartile ranges will be used. Missing data will be analysed and imputation techniques will be used where necessary.

#### Aim 1: longitudinal statistics

Change over time in level of function, activity, HRQoL and satisfaction will be analysed for all continuous outcomes (coronal plane kinematics, hip abductor strength, prosthetic use, mobility level, walking ability, HRQL and prosthesis comfort) and for one categorical outcome (back pain). Generalised estimating equations (GEE) for repeated measurements with an exchangeable correlation structure will be used [59–61]. Back pain will be dichotomised for this analysis into ‘no back pain’ and ‘back pain’ (representing the classes ‘yes, with episodes’ and ‘yes, chronic (daily)’). Continuous outcomes (mean change) and the dichotomised outcome (odds ratio) will be presented with 95% confidence intervals.

#### Aim 2 and 3: prediction models

For the significantly changed outcomes of interest over time (change of coronal plane kinematics, prosthetic use, walking ability, HRQoL and prosthesis comfort) and for stump pain at follow-up, multivariate GEE [59, 61] will be used to examine which potential predictors are of added value in the prediction of these outcomes (Additional file 1). Potential predictors are age, BMI, time from primary amputation to inclusion, cause of amputation, level of amputation, length of the residual limb, baseline hip abductor strength, baseline prosthetic use, baseline mobility level, baseline walking ability and baseline prosthesis comfort. Baseline values of the outcomes of interest will also serve as potential predictors. The multivariate model will be reduced by manually removing predictors with a *p*-value of >0.15 based on the log-likelihood ratio test. Model performance will be assessed by the percentage of explained variance (R^2^) and C-statistics for continuous and categorical outcomes, respectively.

#### Aim 4: causal model for change of back pain

To explore the potential mechanisms in the causal model for change of back pain over time (Fig. 1), the potential determinant (coronal plane kinematics), the potential moderator (stump pain), the potential mediator (hip abductor strength) and the interaction term of the determinant and moderator/mediator will be analysed using GEE.

## Discussion

All assessments included in this study are part of usual care. This is, in our opinion, an advantage because the risk of missing data and loss to follow-up is expected to be low. The Netherlands is a relative small country, resulting in small logistical challenges. This differs from other countries where bone-anchored prosthesis surgery is performed (e.g., Sweden, Germany, Australia). There are a few limitations to our study design. 1) A disadvantage of data collection during usual care is that it may lead to measurement bias. Measurements may have to be performed by a different rater in unforeseen circumstances and the rater is also the treating physiotherapist. Furthermore, the used measurement instruments are rarely gold standard tools. In order to limit measurement bias, we standardised the measuring procedures as described in this study protocol, limited the maximum number of raters to two across all follow-ups and trained the raters. Three of our measurement instruments (Dartfish® angle measurement, Valedo®Motion angle measurement and hip abductor strength test) are developed specifically for this study. Therefore, no information concerning their psychometric properties are readily available, but a reproducibility study concerning these measurements is now ongoing. 2) To increase the compatibility of our study results with other available literature, we have chosen to use the Q-TFA to evaluate prosthesis wearing time (prosthetic use score) and HRQoL (global score) in all patients, regardless of their level of amputation. The Q-TFA is specifically developed for patients with a transfemoral amputation, but the constructs we investigate in this study do not involve specific questions related to the level of amputation that could influence the validity of the results. Furthermore, the Q-TFA is widely used in studies which evaluate bone-anchored prosthesis use, both in patients with a transtibial as with a transfemoral amputation [40]. 3) Walking ability is investigated by patient-reported estimation of the walking distance in daily life. This may be less accurate than using a pedometer or activity tracker, but the results are clinically relevant. Patients’ may overestimate or underestimate, yet this is likely to happen structurally and therefore will not lead to biased results. 4) We have not included a measure investigating back pain intensity. Although back pain frequency is included, the method is too robust to identify more subtle changes, such as back pain intensity, that may also be an important clinical measure of treatment. For example, patients may still experience back pain but to a much lesser degree than before. The prevalence of back pain is high (52–84%) in patients with a lower extremity amputation using a socket prosthesis [21, 62–64]. This study will explore if back pain is a common secondary disability as well in the BAP population and we will examine potential mechanisms for change of back pain over time. Knowledge of these mechanisms can influence the content of rehabilitation programme and future studies may include questions regards to back pain intensity.

In our clinic patients who choose a BAP do this for various reasons, including the expectation to increase their level of function, activity, HRQoL and satisfaction. However, to our knowledge no previous research has been done concerning predictors for outcomes on the level of function, activity, HRQoL or satisfaction. Due to the lack of current evidence, no individualised care can be provided. This causes uncertainty concerning the result of the actual health benefits that a patient is hoping for when choosing for a BAP. This study will be the first to address this topic.

The main inclusion criteria for BAPs in current practice is the presence of socket-related problems. By eliminating these problems, patients aim to overcome limitations in their physical functioning. However, a part of the BAP population suffers from stump pain as result of stoma-related problems, reactivating muscles and reuse of the hip joint for weight bearing [34, 40, 46]. Because stump pain after BAP surgery can also negatively influence physical functioning it is important to quantify the prevalence, the level of stump pain and identify potential predictors. Knowledge concerning these aspects may influence the content of the rehabilitation programme.

In summary, the psychometric properties of some of the chosen measurement instruments are absent and may influence the interpretation of these outcomes. This study is the first to examine coronal plane gait kinematics, hip abductor strength, prevalence of back pain, prevalence of stump pain and level of satisfaction in patients after BAP surgery. This study will provides preliminary insight in associated factors for clinically relevant outcome measures after press-fit BAP surgery. This important knowledge can help patients and professionals alike, to establish realistic expectations of the expected natural course after BAP surgery. In turn, this could potentially result in new inclusion criteria for BAP surgery in the future.

## Additional file

Additional file 1: Prediction models with potential predictors. (DOCX 12 kb)

## Abbreviations

6MWT: 6-Minute walking test
ASIS: Anterior superior iliac spine
BAPs: Bone-anchored prostheses
BMI: Body mass index
GEE: Generalised estimating equations
HHD: Handheld dynamometer
HRQoL: Health-related quality of life
KD: Knee disarticulation
MFC-level: Medicare functional classification level
NRS: Numeric rating scale
PCS: Prosthesis comfort score
QoL: Quality of life
Q-TFA: Questionnaire for persons with a transfemoral amputation
SDC: Smallest detectable change
SIGAM WAP: Special interest group in amputee medicine workgroup amputation and prosthetics
SP: Socket prosthesis
TF: Transfemoral amputation
TT: Transtibial amputation
TUG: Timed up and go
