# Supplementary material for: Long-term outcomes following lower extremity press-fit bone-anchored prosthesis surgery: a 5-year longitudinal study protocol
Source: BMC Musculoskelet Disord. 2016 Nov 22;17:484. doi: 10.1186/s12891-016-1341-z (PMC5120460; doi:10.1186/s12891-016-1341-z)
Supplement: Additional file 1: — Prediction models with potential predictors. (DOCX 12 kb) [file 12891_2016_1341_MOESM1_ESM.docx]

**Supplementary file. Prediction models with potential predictors**

Change of coronal plane kinematics over time compared to baseline= f(time from primary amputation to inclusion, baseline length of the residual limb, baseline coronal plane kinematics, baseline hip abductor strength, baseline walking ability, baseline prosthesis comfort)

Change of prosthetic use over time compared to baseline= f(body mass index, baseline prosthetic use, baseline prosthesis comfort)

Change of walking ability over time compared to baseline= f(age, body mass index, baseline hip abductor strength, baseline walking ability, baseline prosthesis comfort)

Change of health-related quality of life over time compared to baseline= f(baseline prosthetic use, baseline walking ability, baseline health-related quality of life, baseline prosthesis comfort)

Change of prosthesis comfort over time compared to baseline = f(body mass index, time from primary amputation to inclusion, cause of amputation, level of amputation, baseline prosthesis comfort)

Level of stump pain at follow-up= f(time from primary amputation to inclusion, level of amputation, baseline length of the residual limb, baseline hip abductor strength, baseline prosthetic use, baseline mobility level, baseline walking ability)
